# Late Quaternary faulting in the Sevier Desert driven by magmatism

**DOI:** 10.1038/srep44372

**Published:** 2017-03-14

**Authors:** T. Stahl, N. A. Niemi

**Affiliations:** 1 Department of Earth and Environmental Sciences, University of Michigan, Ann Arbor, MI, USA

## Abstract

Seismic hazard in continental rifts varies as a function of strain accommodation by tectonic or magmatic processes. The nature of faulting in the Sevier Desert, located in eastern Basin and Range of central Utah, and how this faulting relates to the Sevier Desert Detachment low-angle normal fault, have been debated for nearly four decades. Here, we show that the geodetic signal of extension across the eastern Sevier Desert is best explained by magma-assisted rifting associated with Plio-Pleistocene volcanism. GPS velocities from 14 continuous sites across the region are best-fit by interseismic strain accumulation on the southern Wasatch Fault at c. 3.4 mm yr^−1^ with a c. 0.5 mm yr^−1^ tensile dislocation opening in the eastern Sevier Desert. The characteristics of surface deformation from field surveys are consistent with dike-induced faulting and not with faults soling into an active detachment. Geologic extension rates of c. 0.6 mm yr^−1^ over the last c. 50 kyr in the eastern Sevier Desert are consistent with the rates estimated from the geodetic model. Together, these findings suggest that Plio-Pleistocene extension is not likely to have been accommodated by low-angle normal faulting on the Sevier Desert Detachment and is instead accomplished by strain localization in a zone of narrow, magma-assisted rifting.

Tectonic and magmatic processes accommodate extension in continental rifts^1^,^2^. The relative contributions of each process are variable in space and time^3^,^4^ with the two processes often being interspersed across or along a broader rift zone, as observed in the East African Rift, New Zealand’s Taupo Volcanic Zone, and the Basin and Range^4^,^5^,^6^,^7^. Over the development stages of continental rifting, protracted extension on normal faults can lead to lithospheric thinning and, in regions of sufficient melt production, magma overpressures that result in the localization of tensile strain, intrusive and extrusive magmatism, and suppressed seismicity^8^,^9^,^10^.

Determining the relative contributions of tectonic and magmatic processes in accommodating extension are essential for interpreting the rheological properties of extensional provinces and attendant seismic hazards. Where extension is accommodated by magmatism and dike intrusion in the upper crust, *non-tectonic, non-seismogenic* normal faults, folds, and fissures will form in response to localized uplift and extension^11^. These faults are incapable of producing >M_W_ 5–6 earthquakes because fault areas are limited by dike dimensions and effective elastic plate thickness in the subsurface (i.e., crustal seismicity is not expected to develop at temperatures >600 °C)^11^,^12^,^13^. Conversely, *tectonic, seismogenic* normal faulting leads to >M_W_ 7 surface rupturing earthquakes and the development of significant topographic relief within the rift^9^,^10^. Tectonic and magmatic faults each have geophysical, geodetic and geomorphic signatures that can be used to discriminate the two mechanisms over decadal to million year timescales (Table 1 and references therein).

The mechanism of normal faulting in the eastern Basin and Range of central Utah (Fig. 1) has been controversial for nearly four decades^14^,^15^,^16^,^17^,^18^,^19^,^20^,^21^. Modern seismicity is focused within the Intermountain Seismic Belt, which encompasses the Wasatch Fault Zone (WFZ) along the easternmost margin of the Basin and Range (Fig. 1A)^22^. Previous analyses of geodetic data demonstrate that the majority of strain is accommodated along the WFZ at the latitude of Salt Lake City^23^,^24^, but there is disagreement as to whether or not significant strain is accommodated across a broader spatial extent at the latitude of the southern WFZ. At this location, discrepant geologic and geodetic slip rates have led to the inference that some extension is accommodated by a low-angle normal fault called the Sevier Desert Detachment (SDD) (Fig. 1A)^25^,^26^,^27^,^28^. Late Pleistocene to Holocene surface-rupturing faults unequivocally extend into the Sevier Desert region, where they displace shorelines associated with major high-stands of pluvial Lake Bonneville^29^,^30^, but the tectonic origin of these faults, and how they relate to potential slip on the SDD, is disputed^28^. The presence of multiple Pliocene to Holocene volcanic centers, aligned along the broadly distributed fault zones in the Sevier Desert, suggests that magmatism may play an important role in accommodating extension west of the southern WFZ^25^,^31^ (Fig. 1B).

Here, we assess the geodetic and geomorphic signature of extension in the eastern Sevier Desert (Fig. 1) and quantify the relative contribution of magmatic and tectonic processes. We use high-precision GPS velocities to constrain elastic dislocation models and to infer the spatial distribution and rates of modern strain accumulation. Field- and remotely surveyed displacements across fault zones constrain the processes (i.e., dike injection or seismic slip) that have driven faulting in this region in the late Quaternary.

## GPS models

The Basin and Range is a key locality for delineating the geodetic signature of diffuse continental extension^23^,^24^,^25^,^26^,^27^, ^32^,^34^,^35^. Nonetheless, the spatial distribution of strain in the eastern Basin and Range of Utah remains unresolved. Studies that employ campaign GPS surveys, with high spatial coverage but low velocity precisions, have generally inferred strain to be localized near the eastern boundary of the Basin and Range^27^. Analyses of continuous GPS data, with high velocity precision but limited spatial coverage, have led to inferences of a broader distribution of deformation in west central Utah, extending as far west as the border with Nevada. These two divergent conclusions have led to differing interpretations of how, and on which faults, strain is actively being accommodated^28^. The localized strain interpretation predicts concentration of most or all of the regional extension on the southern WFZ^26^,^33^, while the broad strain interpretation suggests distributed deformation on multiple Quaternary faults across the WFZ and Sevier Desert, including the SDD^25^,^34^,^35^. These analyses are revisited with velocity estimates derived from decade-long GPS time series collected from 14 continuous GPS stations operated as part of the Plate Boundary Observatory, spanning from the Colorado Plateau in eastern Utah into Nevada.

**Figure 1:**
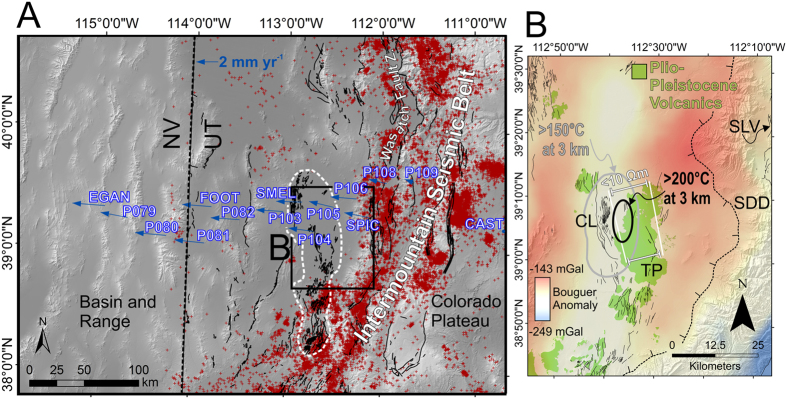
Location and tectono-magmatic context of the study region in the eastern Basin and Range of central Utah. (**A**) Shaded relief map showing the locations of late Quaternary faults (black lines) and seismicity of west-central Utah (red crosses) defining the Intermountain Seismic Belt; GPS stations and velocities used in this study (blue); zone of faulting coinciding with late Cenozoic volcanic centers (white dashed outline); and location of Wasatch Fault Zone. (**B**) Bouguer gravity anomaly map of the eastern Sevier Desert over shaded relief topography, showing coincidence of late Quaternary fault traces (thin black lines) with geophysical and geological features discussed in text. CL: Clear Lake Fault zone. TP: Tabernacle-Pavant Fault zone. SLV: Scipio and Little Valley fault zones. SDD: Surface projection of the Sevier Desert Detachment. Maps created in ArcGIS 10.3 using DEMs from USGS National Elevation Dataset accessed from nationalmap.gov (last accessed September 2016).

GPS velocities in west-central Utah are compared to predicted surface velocities from elastic dislocation models for three potential scenarios of strain accumulation in central Utah: (a) interseismic strain accumulation on a single, dominant fault zone below a specified locking depth (i.e., the WFZ, represented by a creeping edge dislocation in an elastic half-space), (b) interseismic strain accumulation on the WFZ and SDD (two creeping dislocations), and (c) combined strain accommodation by the WFZ (one edge dislocation) and magma-assisted rifting (modeled as a tensile dislocation) in the Sevier Desert. The three model configurations were tested to find the edge and tensile dislocation parameters that best match observed GPS velocities (Table S1) (see Methods and Supplementary information). Faults with ambiguous relationships to deeper structures (e.g. Scipio and Little Valley Faults; Clear Lake-Pavant faults; Fig. 1) were not included in the models, as it is unclear whether these are rooted faults that accommodate interseismic strain^36^. We used Monte Carlo methods to sample fault dip, locking depth, surface position, and interseismic slip rate (or opening rate, in the tensile dislocation case) and used a Bayesian approach to determine acceptable model fits for each strain accumulation scenario (see Methods and Supplementary information)^37^,^38^,^39^.

**Figure 2:**
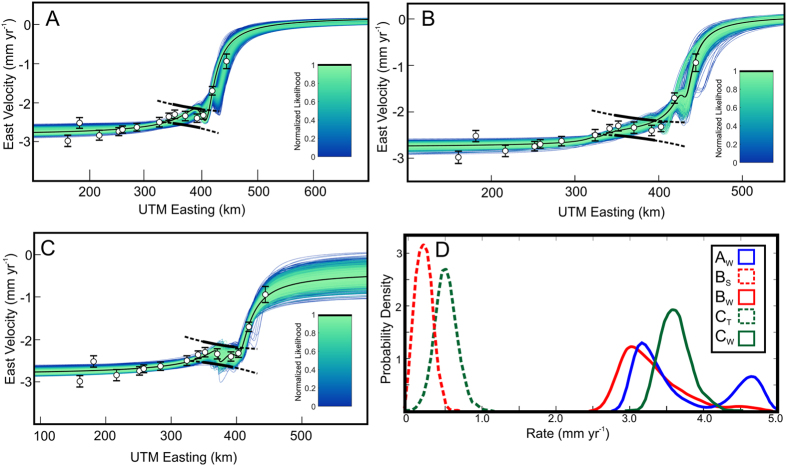
Elastic dislocation model results and observed GPS velocities for three different scenarios. (**A**) Wasatch fault only; (**B**) Wasatch fault and Sevier Desert Detachment; (**C**) Wasatch fault and tensile dislocation. Black lines are the 95% confidence bounds of a linear regression fit to three GPS stations showing contractional strain in the eastern Sevier Desert. Only the best-fitting models in (**C**) reproduce this observation. (**D**) Probability density functions of slip rate predicted from each model, for each dislocation in (**A**–**C**). Legend lettering refers to subfigures of different model runs; subscripts W, S, and T denote distributions for the Wasatch fault, Sevier Desert Detachment, and tensile dislocation, respectively.

Of specific interest in comparing the models was a zone of compression in the eastern Sevier Desert defined by three GPS sites (Fig. 2). Contractional strain observed in geodetic data elsewhere in the Basin and Range has been ascribed to transient deformation associated with historic earthquakes^40^. However, there are no known seismic or paleoseismic events in the Sevier Desert region or on the southern Wasatch fault within the past 500 years^25^ that would produce such deformation.

While all of the elastic dislocation models are capable of producing the broad pattern of GPS velocities, only the model that includes a tensile dislocation is capable of producing the observed distribution of contractional strain across the eastern Sevier Desert (Fig. 2C). The best fitting model parameters for the WFZ in this scenario are an interseismic fault slip rate on the WFZ of 3.42 [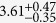, μ and 95% credible interval] mm yr^−1^ on a 29°-dipping fault [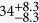] with a 14.3 [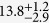] km locking depth (Table S1). These fault parameters are generally consistent with those previously reported for the WFZ^24^,^32^,^41^,^42^, and estimated fault dip is the same as estimates from a shallow seismic section on the southern WFZ^42^. The tensile dislocation parameters are characterized by an opening rate of 0.52 [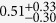] mm yr^−1^ at a depth of 0.8 km [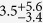] (Table S1).

The modeled positions of the WFZ and tensile dislocation broadly align with mapped faults and volcanic centers, respectively (Fig. 2). We interpret the contractional signal in the Sevier Desert, which is well fit by the addition of a tensile dislocation (Fig. 2C), as being the result of a narrow zone of magma-assisted rifting in the Sevier Desert superimposed on an area of interseismic strain accumulation on the southern WFZ. If this process has been responsible for Sevier Desert extension over 10^2^–10^6^ year timescales, the style of surface deformation should be consistent with dike-induced faulting as observed in other active, magma-assisted rift zones (Table 1 and references therein).

## Field surveys of surface deformation

### Evidence of magma-assisted rifting and dike-induced faulting in the field

Magma-assisted rifts contain seismogenic normal faults of tectonic origin and dike-induced faults of magmatic origin that have distinguishing surface deformation characteristics (Table 1). Dike-induced faults have steep along-strike displacement gradients and often transition from flexural monoclines and fault scarps into tensile fissures^11^. Vertical displacements are symmetric for fault displacements induced by vertical dikes in the subsurface, and the length-scales of far-field displacements are smaller than seismogenic normal faults, owing to typically shorter down-dip fault widths controlled by the dimensions of the dike^43^. Other geomorphic and geologic criteria for discriminating dike-induced faulting include association with cogenetic volcanic rocks, diffuse zones of syn- and antithetic faulting with little net vertical displacement, and subdued topography after 10^3^–10^6^ years of extension (Table 1 and references therein).

### Net vertical displacements across Clear Lake and Tabernacle Hill

**Figure 3:**
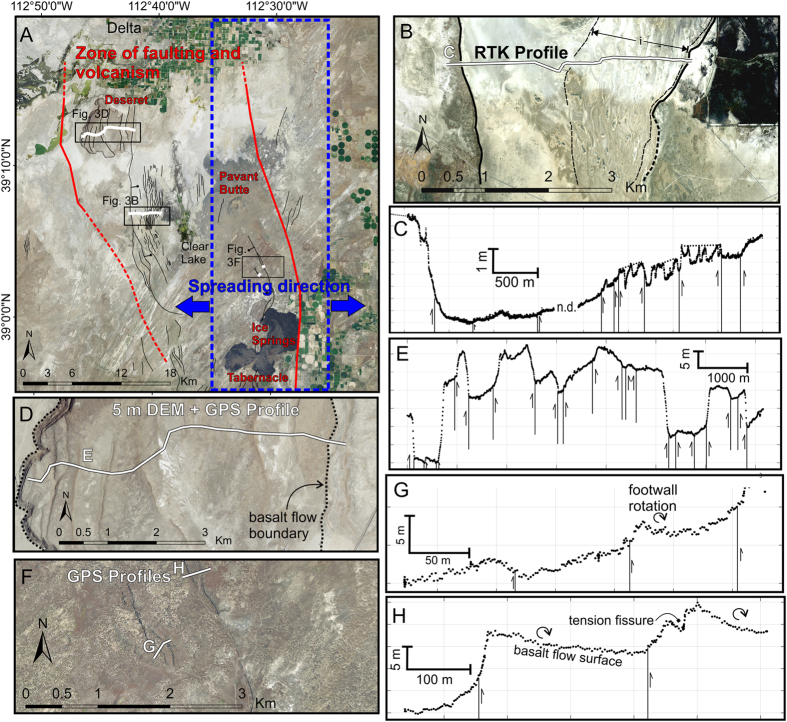
Map and topographic profiles of faulting in the eastern Sevier Desert. (**A**) Overview map showing trend of volcanism (red lines and lettering), Quaternary faults (black lines), topographic profiles (white), and location of spreading center predicted by GPS inversion (blue rectangle and arrows). (**B**) Clear Lake fault aerial imagery and (**C**) topographic profile. All faults are shown as vertical for simplicity. Dashed lines show interpreted hanging wall/footwall slopes where fractures make visual interpretation difficult. (**D**,**E**) Map and topographic profile with Deseret flow surface slope removed. (**F,G** and **H**) Map and topographic profiles across scarps on the Pavant basalt flow (“Devil’s Kitchen fault”^67^). Maps created in ArcGIS 10.3 with aerial imagery from the National Agriculture Imagery Program (NAIP) through Utah’s Automated Geographic Reference Center (AGRC) at https://gis.utah.gov/data/ (last accessed September 2016).

In the eastern Sevier Desert, the Clear Lake and Tabernacle-Pavant fault zones are spatially coincident with Pleistocene volcanic centers and comprise diffuse zones of faults, monoclines and fissures (Figs 1 and 3). The faults displace volcanic rocks, lacustrine sediments of pluvial Lake Bonneville, and the playa surface at Clear Lake. If the displacement on these faults was driven by episodic dike-intrusion in the upper crust rather than displacement on the SDD (as has been previously interpreted^16^,^20^), then these faults would be expected to have diagnostic criteria of other dike-induced faults (see above section and Table 1).

The Clear Lake fault zone consists of two c. 1–4 m high graben-bounding fault scarps with intervening ridges, fractures and faults of <1 m throw that displace the Holocene Clear Lake playa surface^29^,^44^ (Fig. 3). The western bounding fault also forms the edge of the Deseret basalt flow (0.4 ± 0.4 Ma, K-Ar age^45^), and the interior of the flow is displaced by numerous antithetic and synthetic faults with c. 3–15 m displacements (Fig. 3D and E) that pre-date Lake Bonneville (c. 20–15 cal. ka)^30^. The western bounding fault has been active since at least 4.2 Ma, given offsets of a mid-Pliocene basalt observed in seismic reflection profiles^46^, but has not accrued any appreciable topographic relief.

The Clear Lake playa surface can be used as an isochronous datum to measure Holocene displacements across the Clear Lake fault zone. A Real Time Kinematic (RTK) GPS survey was conducted across 10 fault scarps distributed over c. 4 km on the Clear Lake playa (Fig. 3). We calculated cumulative net slip and net throw assuming fault dips of 60 ± 10°, a fault-scarp intersection of 50 ± 10% along the scarp slope distance, and the 95% confidence intervals of linear regression statistics fit to the scarp profile^47^. The calculated cumulative throw across the Clear Lake fault zone is 1.7 ± 2.7 m (1σ) down to the west, which is statistically indistinguishable from zero.

**Figure 4:**
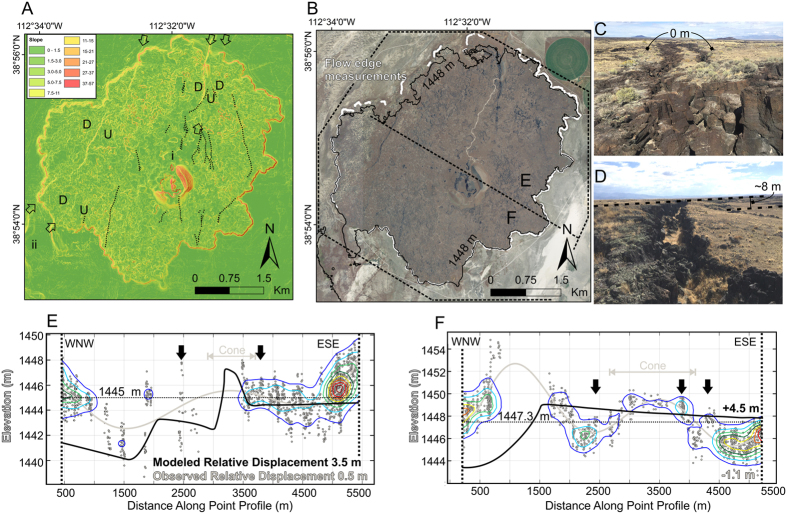
Deformation of the Tabernacle basalt flow. (**A**) Slope map and hillshade showing fault traces (blue arrows), fissures (black dashed lines) and sense of displacement (up and down); (**B**) Aerial photograph showing point measurements of the flow edge (white) in two swaths labeled (**E**) and (**F**); (**C**) Tension fissure with no throw, looking north along the central Tabernacle flow; (**D**) Flexural monocline along strike of (**C**) with combined dilation and 8 m of throw; (**E**) and (**F**) Discordance between observed and modeled displacement field. Point measurements of flow edge elevations (dots) with point density colored (red = densest) to show the variability along transects. Grey line is 7^th^-order polynomial fit to flow edge measurements to show broad wavelength deformation^50^. Black arrows show location of significant fissures along the transect. Black line shows the modeled cumulative fault displacements using Coulomb 3.0 and assuming a 60° fault dip, 5 km down-dip width and a uniform slip distribution. The dotted horizontal line is the original ground surface in the model, plotted at the average flow edge elevation for each transect. Numbers on ESE side of transects in all plots indicate the measured and modeled relative displacements. All maps were created in ArcGIS 10.3 with aerial imagery from the National Agriculture Imagery Program (NAIP) through Utah’s Automated Geographic Reference Center (AGRC) at https://gis.utah.gov/data/ (last accessed September 2016). Digital elevation model (DEM) used to produce (**A**) is from AGRC.

The c. 5–15 km-wide Tabernacle-Pavant fault zone extends from Tabernacle Hill to Pavant Butte and consists of swarms of fissures, faults, fault line scarps, and monoclines (Figs 1B and 4). At Tabernacle Hill, the basalt flow surface (17.3 ± 0.3 cal. ka^29^,^48^) is variably deformed by N-NE-striking faults, monoclines (some with basal compressional ‘push-up’ structures^49^), and fissures (Fig. 4). Vertical displacements on faults reach up to c. 8 m locally, but have steep along-strike displacement gradients and grade into tension fissures or terminate within 1 km of the maximum displacements. Vertical displacements die out rapidly perpendicular to the faults, implying short down-dip fault widths (Fig. 4).

We surveyed fault scarps in the Tabernacle-Pavant fault zone using RTK GPS and took >2500 elevation measurements of the Tabernacle basalt flow surface perimeter using a 5 m DEM to test the observed distribution of displacements vs. those expected for a fault soling into the SDD at 5 km depth. The perimeter of the basalt flow surface was used as a paleo-horizontal datum because previous mapping indicates that the elevation of the circular flow surface was controlled by the lake level into which the basalt erupted^29^. The data show that flow surface perimeter elevations are approximately the same on either side of the flow, within uncertainty, despite large on-fault throws and broad c. 2–3 km wavelength regions of uplift and subsidence across the flow surface (graphically represented by 7^th^ order polynomial fit to elevations, following procedure of ref. 50) (Fig. 4). The expected net coseismic displacement field for faults with a 5 km down-dip width (Figs 4G and H) deviate from our observations by c. 3–5.7 m over the same distance. Thus, while fault throw here is apparently asymmetric (i.e. throw on antithetic faults is not entirely compensated by synthetic faults), this displacement is recovered over a short distance (Fig. 4).

Cumulative horizontal and vertical displacements across antithetic and synthetic normal faults in the hanging wall of a low-angle detachment should sum to yield the net displacement vector on the underlying detachment^51^. The net vertical displacements across the Clear Lake and Tabernacle faults in the Sevier Desert region are indistinguishable from zero (e.g., Fig. 4). This observation is consistent with faulting caused by shallow dike emplacement, but inconsistent with displacement on a dipping detachment fault like the SDD and associated tectonic faulting^43^.

### Comparison of predicted and observed extension rates

**Figure 5:**
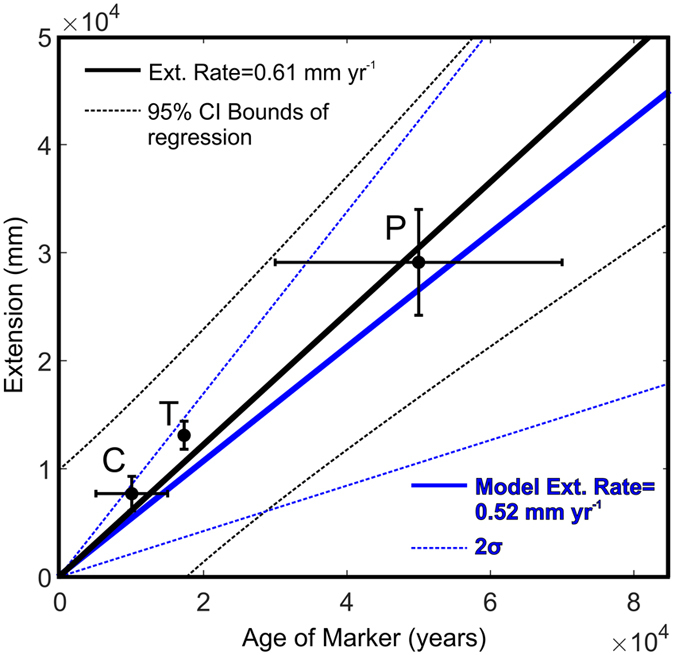
Age of marker vs. net extension for the Clear Lake and Tabernacle-Pavant fault zones. The best-fitting line between Clear Lake (C), Tabernacle Hill (T) and Pavant (P) has a slope of 0.61 (when forced through intercept = 0), which matches well with the rate predicted by GPS inversion (blue lines).

To further test the hypothesis that faulting in the eastern Sevier Desert is non-tectonic, we compare the magnitude of horizontal extension across the fault systems with extension rates resolved on a tensile dislocation from the inversion of GPS data (Fig. 2C). We calculated the net horizontal extension across fault scarps at three locations with reasonable age control and found the best-fit regression of displacement vs. age (Fig. 5) (see Methods and Supplementary information). The results show that the extension rate resolved on faults at Clear Lake, Pavant Butte, and Tabernacle Hill is 0.61 mm yr^−1^ over the last c. 50 ka, which is consistent with the opening rate of the tensile dislocation (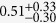 mm yr^−1^) in the preferred geodetic solution (Figs 2C and 5). By comparison, the range of slip rates for strain accumulation on the SDD is significantly less (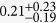 mm yr^−1^) (Fig. 2B) than the calculated geologic extension rate.

## Modes of extension in west-central Utah

### Slip on the Wasatch Fault and extension in the Sevier Desert

GPS data from the eastern Basin and Range can be used to constrain the mechanisms of modern strain accumulation. All of the strain accumulation scenarios that we tested (Fig. 2) produce WFZ parameters (i.e., slip rates, locking depths, and dips) consistent with previous geodetically-derived estimates^23^,^24^,^25^,^26^,^33^,^34^,^35^, although not with recent combined geodetic-geologic inversions^52^. However, the distribution of contractional strain observed across the eastern Sevier Desert in the geodetic data is only explained by modelling a tensile dislocation, rather than an edge dislocation, in the Sevier Desert (Fig. 2). In addition, surface deformation in the Sevier Desert is not consistent with tectonic faults soling into a detachment at depth (Table 1, Figs 3 and 4). The geologic extension rate for the last c. 50 ka across this region (Fig. 5) is similar to the geodetic extension rate for an opening tensile dislocation, but inconsistent with the range of acceptable extension rates inferred for an actively slipping SDD. Taken together, we propose a model of extension in the eastern Sevier Desert in which localized crustal extension is accommodated in the uppermost crust by periodic volcanism and dike intrusion, superimposed on broad extension dominated by slip on the southern WFZ.

### Implications for seismic hazard and the Sevier Desert Detachment

Rupture of a low-angle normal fault^53^ and on populations of high angle faults that sole into such a fault at depth^54^ is capable of producing earthquakes significantly larger than M_W_ 7, whereas dike-induced earthquakes do not usually exceed c. M_W_ 5.5 and are commonly smaller^11^. This magnitude difference equates to a minimum 200 times difference in seismic moment release and results in vastly differing estimates of maximum earthquake magnitudes and seismic hazard. Our data suggest that the SDD is not actively accumulating strain and is therefore unlikely to generate large earthquakes.

Over 80% of the extensional strain in our preferred model is resolved on a gently- to moderately-dipping WFZ at depth, implying that the bulk of the modern seismic hazard still resides in the area around the southern WFZ. The geodetic WFZ slip rates we derived are an order of magnitude larger than geologic slip rates for the southern WFZ segments^28^,^52^. There are several possible reasons for this discrepancy, including an under-estimation of geologic rates due to unrecognized, distributed hanging wall faulting, or time-variable slip rates with increased modern interseismic loading^28^. While the slip rate discrepancy on the southern WFZ is not the focus of this study, our results do discount interseismic strain accumulation across the SDD as a viable mechanism for accommodating the regional geologic moment deficit^28^.

### Magma-assisted rifting in the Sevier Desert

Magma-assisted extension in the eastern Sevier Desert is consistent with existing geophysical and geochemical data from the region (Fig. 1). The aseismicity of the eastern Sevier Desert (Fig. 1A) is consistent with other regions of high geotherms and magma supply, such as in segments of the East African Rift^10^ and the eastern Snake River Plain, but at odds with observations of microseismic activity associated with active detachment faults^37^,^55^,^56^. The broader Sevier Desert region (white dashed lines, Fig. 1A) is also distinguished from surrounding regions by relatively high heat flow, low lithospheric thickness, and shallow Late Cenozoic intrusions^57^,^58^.

Subsurface temperatures near the Clear Lake fault zone at Pavant Butte reach 200 °C at c. 3 km depth, where a symmetrical, negative Bouguer gravity anomaly approximately coincides with a vertical zone of low resistivity (<10 Ωm) extending to 6–18 km depth (Fig. 1B)^59^,^60^,^61^. These observations, along with the predominance of subalkaline volcanism in the eastern Sevier Desert, have previously been proposed to be due to the initiation of magmatic rifting and/or geothermal fluid flow near the Basin and Range-Colorado Plateau transition zone^62^.

On the other hand, a wealth of seismic reflection data in the region^63^ and aeromagnetic surveys^64^ have not yielded compelling evidence for the existence of widespread shallow intrusions in the Sevier Desert. The lack of seismic reflection evidence for dyking is perhaps not surprising given the difficulty seismic reflection surveys face in detecting narrow, vertical discontinuities. Likewise, a lack of magnetic anomalies associated with proposed dikes (under the Clear Lake playa, for instance) is not unexpected given the resolution of the aeromagnetic surveys and distribution of late Cenozoic volcanic units, both buried and exposed, in defining magnetic ‘relief’^64^.

The evidence presented in this study suggests that Basin and Range extension is being accommodated by magmatism in the Sevier Desert and has been for at least the last 50 kyr (Fig. 5). If the onset of basaltic volcanism in the Sevier Desert during the Pliocene is an indication of magma-dominated extension, then this process may have been active since c. 6–3 Ma^65^. Such an interpretation diminishes the likelihood of post-Miocene slip on the SDD^63^, but does not directly bear on whether this fault contributed to pre-Pliocene extension, as has been inferred by several thermochronology studies^17^,^66^ and is supported by balanced cross-sections^21^,^67^. It is possible that magma-assisted rifting has supplanted low-angle normal faulting as the dominant mode of extension in the eastern Sevier Desert through time, as has been observed in other evolving extensional systems globally^8^. This spatiotemporal superposition of magmatic on tectonic extension observed along the Wasatch Front and in the Sevier Desert warrants investigation in other extensional regimes where the mechanism of faulting bears directly on regional seismic hazard.

## Methods

### GPS velocities

Continuous GPS velocities were obtained from 14 Plate Boundary Observatory (PBO) stations in west-central Utah (https://www.unavco.org/data/gps-gnss/derived-products/derived-products.html, last accessed June 2016). Occupation period of the sites ranges from 7–18 years and in all cases the standard deviation of the Easting velocity (σ) is ≤0.2 mm yr^−1^, with σ_avg_ = 0.12 mm yr^−1^. GPS velocities are in the NAM08 reference frame. Position offsets due to earthquakes and equipment changes were estimated following Geodesy Advancing Geosciences and Earthscope (GAGE) processing protocols; outliers (e.g., due to excess snow) were removed from time series prior to calculating velocities (see GAGE processing and data analysis plan at https://www.unavco.org/data/gps-gnss/derived-products/derived-products.html, last accessed June 2016). We obtain net E-W extension rates across the Nevada-central Utah transect at c. 39.5°N of c. 2.5–3 mm yr^−1^, which is similar to previously published data^25^,^33^.

### Elastic Dislocation Models

Observed GPS velocities were used to constrain fault and tensile dislocation positions, geometries and slip rates using elastic dislocation models, following published procedures^33^,^37^. Faults were modeled as edge dislocations of infinite depth and width in an elastic half space described by a surface location, locking depth, dip, and slip rate^39^. Magmatic deformation was modeled as an infinite tensile dislocation in an elastic half space described by a surface location, locking depth, and opening rate. Monte Carlo simulations of surface displacements were generated for 10^8^ combinations of randomly selected fault and tensile dislocation parameters and compared to the observed GPS velocities. The *likelihood* that a set of model parameters describes the observed GPS velocities was defined by the criterion *e*^−0.5*L2*Norm*^. Model parameters that provide an acceptable fit to the observed velocities were retained, based on this criterion, using standard Bayesian methods.

### RTK and GPS Surveys

Real Time Kinematic (RTK) surveys were conducted with a Trimble R8 rover and GPS surveys with a handheld Garmin GPSMap 64st, respectively. RTK positions are based solely on geodetic positioning, and were corrected on the fly with virtual reference stations (VRS) using Utah’s TURN network. Vertical accuracy of positions is <10 cm and precision within any single transect is estimated to be ≤1 cm. GPS survey horizontal positions are based on geodetic position, while vertical positions were obtained with a barometric altimeter calibrated to known spot elevations. Vertical precision within any transect is estimated to be <1 m; pressure variations due to weather were observed, noted and any discrete jumps in the dataset were manually removed. The recording interval of RTK and GPS surveys were 0.5 m and 2 seconds (c. 1.5 m ground distance in flat terrain), respectively.

### Fault Heave, Throw, and Net Slip

Survey points were projected onto a plane perpendicular to local fault strike and regression statistics were calculated for linear fits to topographic profiles of the hanging wall, footwall, and scarp. Best-fit lines fit to irregular scarps (e.g., fold scarps, or those with inferred or observed components of dilation on the fault) were treated the same as fault scarps proper, as there is evidence that the fault breaches the scarp in similar structures formed in basalt^49^,^68^. Net fault slip was then calculated via Monte Carlo simulation over 25,000 iterations, using normal distributions defined by the mean and standard error of inputs (slope of the hanging wall, footwall, and scarp)^47^. Normal distributions were also used as inputs for fault dip and location of the scarp-fault plane intersection, using standard values for Basin and Range normal faults that are consistent with local subsurface observations (see text for details)^20^,^47^,^69^. Faults were considered to be entirely dip-slip. Each net slip value was then converted to constitutive throw and heave components using fault dip and, where reported, summed across other faults in the transect. Uncertainties of the throw and heave distributions are reported as 95% confidence intervals.

## Additional Information

**How to cite this article**: Stahl, T. and Niemi, N. A. Late Quaternary faulting in the Sevier Desert driven by magmatism. *Sci. Rep.* **7**, 44372; doi: 10.1038/srep44372 (2017).

**Publisher's note:** Springer Nature remains neutral with regard to jurisdictional claims in published maps and institutional affiliations.

## Supplementary Material

Supplemental Information

## Figures and Tables

**Table 1:** Criteria for determining magmatic vs. tectonic rifting.

Spreading center with episodic dike-induced faulting criterion	Tectonic faulting/LANF criterion	Relevant dataset/figure
*Geodetic*		
Dike-induced local extension and subsidence within compressional stress ‘shadow’^43^	Broad wavelength extension across locked fault^37^,^39^	Figs 2 and 5
*Geologic/Geomorphic*		
Demonstrated or inferred association with cogenetic volcanic rocks; faults buried in near-vent areas by cogenetic volcanic rocks^11^	No particular association with any rock type	Figs 1,3 and 4
Diffuse belts of faulting several kilometers wide^11^,^49^	Typically 10^–1^ to 10^0^ kilometer-wide fault zones at surface (depending on fault maturity and basement depth)	Figs 1,3 and 4
Graben or zones of noneruptive fissures symmetrical about an eruptive fissure^11^	Pattern and location of faulting form irrespective of eruptive centers; some volcanism may be preferentially channeled along faults	Figs 1,3, 4
Maximum fault widths equal to or slightly greater than depth of dike/extension^43^	Faults extend to regional detachments or seismogenic depths	Fig. 4
Tensional fissures are most abundant feature with little net vertical displacement across the graben^11^,^49^	Normal faults in unconsolidated sediments are most abundant feature	Fig. 4
Monoclinal flexures and vertical normal faults common in basalt^11^,^49^	Vertical normal faults and broad monoclines less common than normal fault scarps^11^	Figs 1 and 4
Vertical displacements vary abruptly along strike, commonly grading into monoclines or tension fissures^11^,^70^	Vertical displacements typically vary systematically along segment strike^71^,^72^	Figs 3 and 4
Subdued topography after 10^6^ year extension^9^,^11^	Development of footwall ranges and/or metamorphic core complexes and hanging wall basins after 10^6^ year extension	Figs 1,3 and 4
*Geophysical*		
Association with symmetrical geophysical anomalies^11^	Association with asymmetric geophysical anomalies	Fig. 1
Suppressed or episodic, variable depth seismicity associated with dike-intrusion^1^,^9^	Recurring regional seismicity at seismogenic depths or on high-angle splays soling into LANF^55^	Fig. 1
