# Supplementary material for: Late Quaternary faulting in the Sevier Desert driven by magmatism
Source: Sci Rep. 2017 Mar 14;7:44372. doi: 10.1038/srep44372 (PMC5349561; doi:10.1038/srep44372)
Supplement: Supplemental Information [file srep44372-s1.pdf]

Late Quaternary faulting in the Sevier Desert driven by magmatism

Supplementary Information

Tim Stahl[1] and Nathan Niemi[1]

[1]Department of Earth and Environmental Science, University of Michigan

1100 N. University Avenue

Ann Arbor, MI 48109

Included in Supplementary Information:

- Supplementary Methods
    - Determining uncertainty in extension and ages
    - GPS inputs
- Supplementary Figures
    - Figure S1
    - Figure S2
- Supplementary Table
    - Table S1
- Supplementary References

Supplementary Methods

Uncertainty in Ages and Extension

This section describes the data and uncertainties presented in Figure 5. The ages of the Tabernacle Hill basalt flow, Clear Lake playa, and Pavant basalt flow have varying degrees of uncertainty. The Tabernacle Hill flow has been dated indirectly using ^{14}C of tufa on the eastern edge of the flow[1], which yielded an age of 14320 ± 90 ^{14}C years. From this age and other geomorphic and geologic observations (e.g., presence of pillows and palagonite-altered tuff, stratigraphic relationships with Lake Bonneville marl), Oviatt [1] proposed the Tabernacle Hill flow erupted into Lake Bonneville during the Provo high-stand. Goehring, et al. [2] used a 1σ calibrated age of 17.3 ± 0.3 ka BP for the flow using calibration of Reimer, et al. [3] and returned reasonable ^{3}He production rates consistent with reference rates. As such, we used the age range given in Goehring, et al. [2].

The Clear Lake playa sediments have not been directly dated and thus a conservative age uncertainty (10.0 ± 5.0 ka) was assigned. It is likely that the playa surface itself is continuously being deposited and redeposited in response to wetting/drying cycles and eolian influx. However, some studies have noted that lacustrine and fluvial sediments can be found in the shallow subsurface (<10 m)[4,5], which likely relate to retreating Lake Gunnison and overlying sandy gravel deposition by the Beaver River around 11-7 ka BP[5,6]. The playa can be no older than the overflowing stage of Lake Gunnison (c. 13.1 cal. ka BP). We therefore consider the age of 10.0 ± 5.0 ka a conservative surrogate for formation of the playa surface. We acknowledge that if the scarps formed over multiple deformation cycles between resurfacing events on the playa, then extension rate presented in Fig. 5 is a minimum. For instance, McBride, et al. [5] interpreted a larger vertical offset at depth on the western-most scarp than is observed at the surface based on description of hand-augered sediments. However, it is unclear if this represents deposition against a pre-existing scarp or ongoing displacement of older units.

The Pavant basalt has a wide range of reported values (c. 30 ka to 220 ka)[7-9] from K-Ar ages, with uncertainties as large or larger than the ages themselves. The best data come from Hoover [9], who argues for a maximum age of 128 ka for the older of two flows; Hoover [9] and Johnsen [10] report a range of 30-70 ka for the flow where our RTK profiles were taken. As such, we used an age of 50 ± 20 ka in Fig. 5.

Extensions (Fig. 5, y-axis) were reported as mean and 1σ of the Monte Carlo simulation output (described above and in main text).

GPS Inputs

This section describes the use of preliminary models to define input distributions for the GPS inversion in Fig. 2 and Table 2. Initial model runs for geometry 3 (Wasatch + tensile dislocation) revealed that only a limited range of surface projections allowed enough acceptable model fits (>1000 n). To increase the number of acceptable model runs, we refined the range of allowable Wasatch fault dips (35 ± 5) based on output from initial model runs, the other model geometries, and geologic studies[11]. Additionally, we refined the range of allowable surface projections to normal distributions with less conservative uncertainty based on (i) the known location of the Wasatch surface projection; and (ii) the range of initial outputs for the Wasatch fault and tensile dislocation in the Sevier Desert. As the initial range of UTM surface projections aligns roughly with the zone of faulting and volcanism in question, this refinement was considered reasonable.

Supplementary Figures

Pair Plots

This section shows density pair plots for the model outputs in Fig. 2.

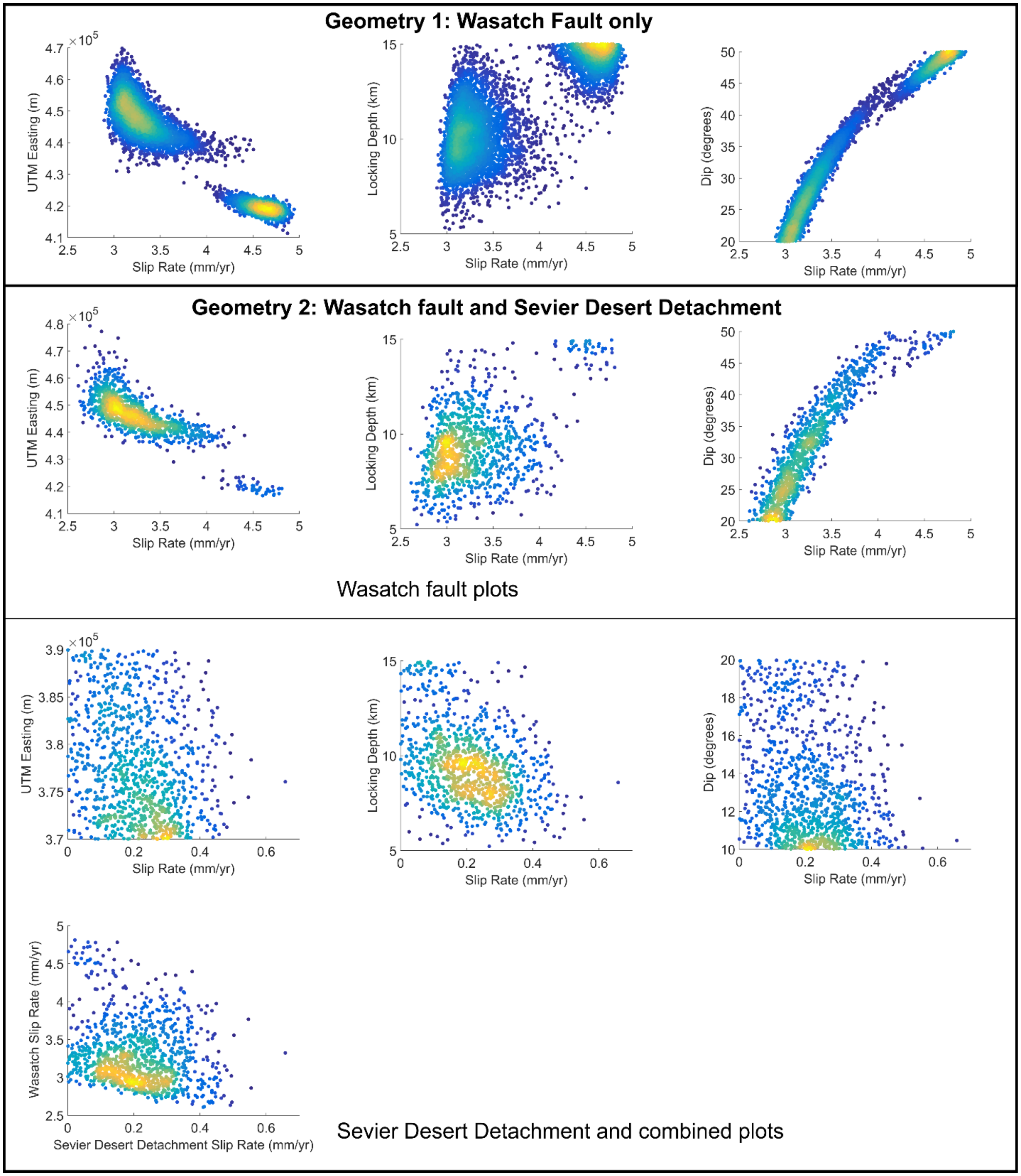

Figure S1: Pair plots for model geometries 1 and 2, colored by density of points.

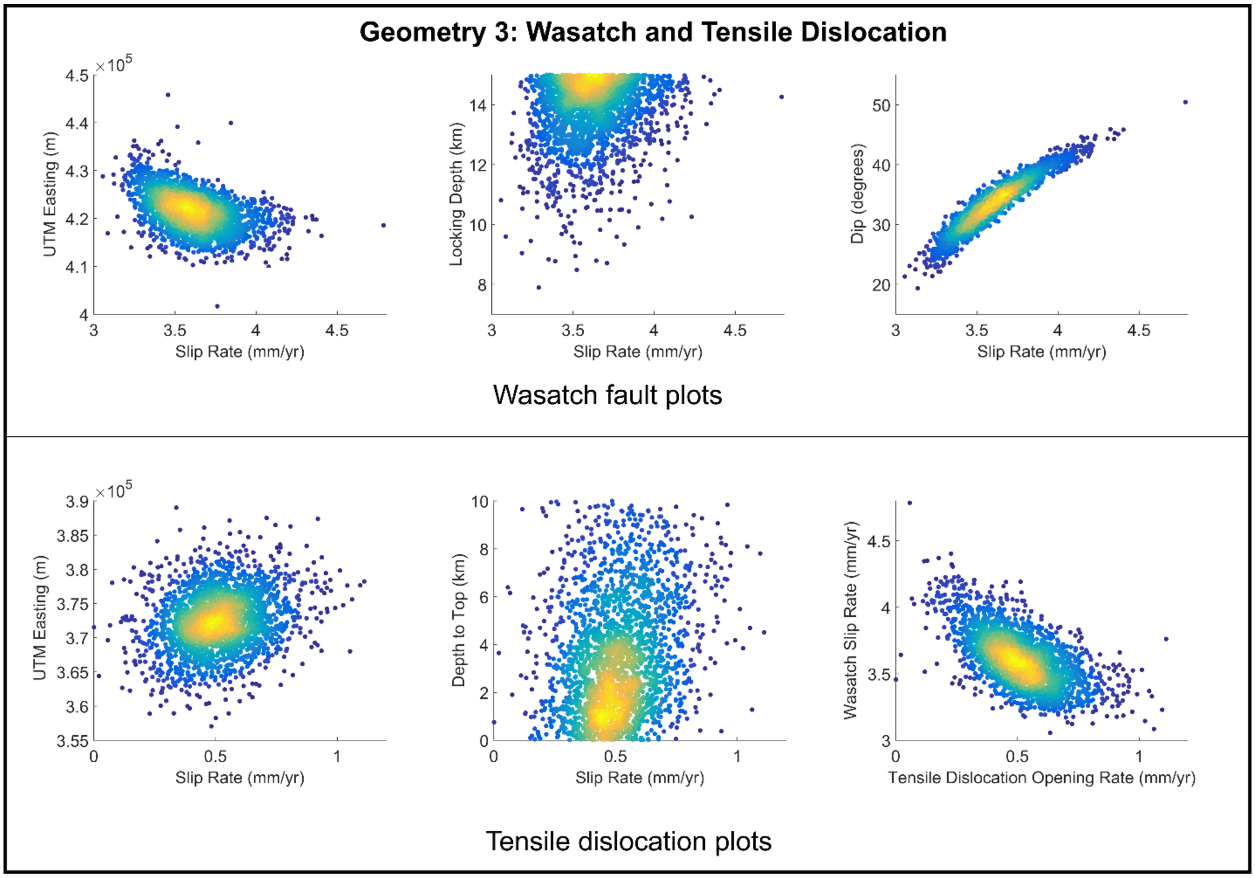

Figure S2: Pair plots for geometry 3, colored by density of points.

Supplementary Table

Table S1: Range of values and model results for GPS inversion.

**Model Scenario and Parameters**	**Range of Values***	**Model Results****
Wasatch Only		
Surface Position (UTM Easting)†	400000–500000	418616 [439168^{+21669}_{-22219}] m
Locking Depth (km)	5–15	14.9 [$11.4^{+3.5}_{-4.1}$]
Dip	20–50°	49.2 [34^{+15}_{-14}]
Slip Rate (mm yr^{-1})	2–6	4.76 [$3.39^{+1.1}_{-0.7}$]
Wasatch[1] and Sevier Desert Detachment[2]		
Surface Position	400000–500000[1]<br><br>365000–395000[2]	445487 [445821^{+17265}_{-26581}]<br><br>370017 [377763^{+11429}_{-7557}]
Locking Depth	5–15	7.88 [$9.43^{+5.07}_{-3.30}$]
Dip	20–50[1]<br><br>5–20[2]	29 [32^{+16}_{-12}]<br><br>10 [13^{+6}_{-3}]
Slip Rate	2–6[1]<br><br>0–6[2]	3.03 [$3.29^{+1.21}_{-0.55}$]<br><br>0.27 [$0.21^{+0.23}_{-0.19}$]
Wasatch[3] and Tensile Dislocation[4]		
Surface Position	420000 ± 10000[3]<br><br>370000 ± 5000[4]	426316 [421514^{+8355}_{-7751}]<br><br>371534 [372423^{+8664}_{-8754}]
Locking Depth<br><br>Depth	5–15[3]<br><br>0–10[4]	14.3 [$13.8^{+1.2}_{-2.9}$]<br><br>0.86 [$3.5^{+5.6}_{-3.4}$]
Dip	35 ± 5[3]<br><br>90[4]	29 [34^{+8}_{-8}]<br><br>90
Slip Rate	0–6[3]<br><br>0–2[4]	3.42 [$3.61^{+0.47}_{-0.35}$]<br><br>0.52 [$0.51^{+0.33}_{-0.30}$]

*Uniform distributions are denoted by ranges between maxima and minima; normal distributions are denoted by mean ± 2σ

**Values in brackets denote mean and 95% credible interval

Supplementary Information References

1 Oviatt, C. G. Quaternary geology of the Black Rock desert, Millard County, Utah. *Utah Geological Survey Special Study* **73,** 27 p. (1991).

2 Goehring, B. M. *et al.* A reevaluation of in situ cosmogenic 3He production rates. *Quaternary Geochronology* **5**, 410-418, doi:10.1016/j.quageo.2010.03.001 (2010).

3 Reimer, P. J., Baillie, M. G. L., Bard, E. & Bayliss, A. IntCal04 terrestrial radiocarbon age calibration, 0-26 cal kyr BP. *Radiocarbon* **46**, 1029-1058 (2004).

4 Bucknam, R. C., Anderson, R.E. Map of fault scarps on unconsolidated sediments, Delta , 1 degree by 2 degree quadrangle, Utah. Report No. 79-366, 22 (USGS, 1979).

5 McBride, J. H. *et al.* Neotectonics of the Sevier Desert basin, Utah as seen through the lens of multi-scale geophysical investigations. *Tectonophysics* **654**, 131-155, doi:10.1016/j.tecto.2015.05.007 (2015).

6 Oviatt, C. G. Quaternary geology of part of the Sevier Desert, Millard County, Utah. *Utah Geological and Mineral Survey Special Study* **70,** 46 p. (1989).

7 Best, M. G., McKee, E. H. & Damon, P. E. Space-time-composition patterns of late Cenozoic mafic volcanism, southwestern Utah and adjoining areas. *American Journal of Science* **280**, 1035-1050, doi:10.2475/ajs.280.10.1035 (1980).

8 Condie, K. C., Barsky, C.K. Origin of Quaternary basalts from the Black Rock Desert region, Utah. *Geological Society of America Bulletin* **83**, 333-352 (1972).

9 Hoover, J. D. Periodic Quaternary volcanism in the Black Rock Desert, Utah. *BYU Geology Studies* **21**, 3-73 (1974).

10 Johnsen, R. L., Smith, E.I., Biek, R.F. Subalkaline volcanism in the Black Rock desert and Markagunt Plateau volcanic fields of south-central Utah. Utah Geological Association Publication **39** , 109-150 (2010).

11 Smith, R. B. & Bruhn, R. L. Intraplate extensional tectonics of the Eastern basin-Range: Inferences on structural style from seismic reflection data, regional tectonics, and thermal-mechanical models of brittle-ductile deformation. *Journal of Geophysical Research: Solid Earth* **89**, 5733-5762, doi:10.1029/JB089iB07p05733 (1984).
